# Allelic diversity of the pharmacogenes CYP2D6 and CYP2C19 in Māori from Te Tairāwhiti, Aotearoa New Zealand

**DOI:** 10.3389/fgene.2026.1668409

**Published:** 2026-02-27

**Authors:** Leonie Hitchman, Te Whetu Aarahi Kerekere, Allison L. Miller, Elizabeth Goodin, Caroline Koia, Huti Watson, Frances King, Stephen P. Robertson, Phillip Wilcox, Martin A. Kennedy

**Affiliations:** 1 Department of Pathology and Molecular Medicine, University of Otago, Christchurch, New Zealand; 2 Department of Womens and Childrens Health, University of Otago, Dunedin, New Zealand; 3 Ngāti Porou Oranga, Gisborne, New Zealand; 4 Department of Mathematics and Statistics, University of Otago, Dunedin, New Zealand

**Keywords:** CYP2C19, CYP2D6, diversity, drug metabolism (human and animal), drug response, indigeneous, nanopore sequencing

## Abstract

CYP2C19 and CYP2D6 are two important pharmacogenes responsible for metabolising a wide range of medications. Both genes exhibit a high level of variation, which can lead to variable activity of the enzymes they encode, with risks of adverse drug reactions and treatment failure. Understanding this variation is therefore of great importance, but the full extent of variability in these genes is not yet documented, particularly for understudied populations. We employed targeted nanopore sequencing to identify genetic variants within *CYP2C19* and *CYP2D6* for a group of Māori individuals, largely affiliated with the Ngāti Porou iwi from Te Tairāwhiti (Gisborne), Aotearoa New Zealand. 135 *CYP2D6* and 73 *CYP2C19* genotypes were sequenced, with metaboliser phenotypes inferred for the majority of participants. CYP2D6 normal metabolisers make up 54% of the cohort and 45% of the cohort are CYP2C19 intermediate metabolisers. Nearly 20% had an uncertain CYP2D6 activity due to the prevalence of CYP2D6*71, which is of unknown functional impact. Understanding the extent of variation in these genes should contribute to equitable application of pharmacogenetic testing in Aotearoa New Zealand.

## 1 Introduction

Pharmacogenetics is the study of genes which impact drug response. It is now clear that increased understanding of these genes and application of this knowledge will help to reduce instances of adverse drug reactions and treatment failure (Relling et al., 2020; Pirmohamed, 2023). Two genes for drug metabolising enzymes that show considerable functional variability and for which there is strong evidence for clinical utility are *CYP2D6* and *CYP2C19* (Nofziger et al., 2020; Taylor et al., 2020; Botton et al., 2021). The gene encoding liver enzyme cytochrome P450 2D6 (*CYP2D6)* has been extensively studied, and the protein it encodes is responsible for metabolising approximately 25% of commonly prescribed medications. *CYP2D6* displays extensive polymorphic variation (Nofziger et al., 2020), and indeed is amongst the most variable genes in the genome. *CYP2C19* is also a well-studied, relatively polymorphic pharmacogene encoding the principal metabolising enzyme for several clinical drugs ranging from antidepressants and proton pump inhibitors to antiplatelet medicines (Ionova et al., 2020; Botton et al., 2021). Despite the abundance of research on these genes, and their clear clinical relevance, the range of *CYP2D6* and *CYP2C19* variation in many indigenous or other understudied populations is still unknown.

The indigenous people of Aotearoa New Zealand, known as Māori, descend from their Eastern Polynesian ancestors who first settled here in ∼1,250–1,300 AD (Gosling and Matisoo-Smith, 2018). Modern day Polynesians trace their ancestry back to South East Asia with migration into the Pacific region during the mid-Holocene era some 3,000–5,000 years ago (Gosling and Matisoo-Smith, 2018). The Māori ethnic population currently represents over 17% of the population in Aotearoa New Zealand, but detailed analysis of variation in key pharmacogenes in Māori is lacking (Helsby, 2016).

We previously investigated the *CYP2D6* genotypes of 202 Polynesian individuals, using nanopore sequencing to analyse a 6.6 kb amplicon containing the entire gene, assigning star alleles based on direct haplotype information, and discovering several previously unreported variants (Hitchman et al., 2022). In addition, we determined that an allele called CYP2D6*71, defined by rs118203758 (G42E) and reported to be very rare in Europeans and other ethnicities, occurred with a frequency of close to 10% in this population.

Our current study expands on this previous work by analysing both *CYP2D6* and *CYP2C19* in a group of individuals of Māori descent, mostly affiliated with the iwi (tribe) Ngāti Porou, from the Eastern North Island region of Te Tairāwhiti (Gisborne).

## 2 Materials and methods

Ethical approval for this study, ‘Rakeiora: A Pathfinder for Research into Genomic Medicine’ (Ethics ref: 20/STH/185), was obtained from Southern Health and Disability Ethics Committee. 151 individuals were recruited by Ngāti Porou Hauora in Te Tairāwhiti (Gisborne Region, New Zealand) and consented to be part of this study. This study is one of several projects under the Rakeiora banner that aims to develop acceptable and appropriate research methods and practices pertaining to precision medicine for Māori communities (Rye, 2025).

Materials and methods used to analyse *CYP2D6* have been described previously (Liau et al., 2019; Hitchman et al., 2022). Briefly, a 6.6 kb *CYP2D6* amplicon encompassing the entire gene including upstream and downstream non-coding regions was generated by polymerase chain reaction (PCR). Additional primers were included to amplify a 3.5 kb amplicon if a gene duplication or deletion was present (Johansson et al., 1996). The 6.6 kb amplicon for each sample was subsequently purified using magnetic beads before a barcoded, pooled (≤96 sample) library was prepared. Approximately 90 ng (∼20 fmoles) of library was loaded on a FLO-FLG-001 flongle (R9.4.1) on the GridION X5 and run for a maximum of 24 h.

*CYP2C19* is a much larger gene (∼93 kb), and in order to analyse variation within the coding regions, we generated six PCR amplicons ranging from 3.5 to 7.1 kb in size (Supplementary Table S1). These amplicons encompassed all nine exons and the promoter region. The initial primers covering exon 7 proved inconsistent, therefore a second primer pair was designed and used in such cases, termed exon 7b (Supplementary Table S1). The amplicons were purified using magnetic beads (MagBio Genomics Inc., Gaithersburg, Maryland, USA) and pooled by participant for barcoding. Up to 12 participants were sequenced per run. The pooled library was prepared using approximately 180 ng (∼40 fmoles) of library and sequenced on a FLO_MIN106 MinION flow cell (R9.4.1) on the GridION X5 for ∼1–3 h. Separate multiplex libraries and separate flow cell runs were used for each of the genes in most cases, although for some runs amplicons from both genes were multiplexed and run together.

All barcodes with a read depth >100x across the amplicon were taken forward for analysis via a previously designed pipeline (Graham et al., 2020) and variants were manually checked with IGV and compared with the relevant PharmVar reference sequence, as previously described (Hitchman et al., 2022). Sanger sequencing was used to validate potentially novel *CYP2D6* variants using the primers as described (Hitchman et al., 2022) and phenotypes were assigned with reference to published activity scores (Caudle et al., 2020; Lee et al., 2022).

Established *in silico* tools based on evolutionary conservation used for predicting functional impact of coding variants do not perform well with pharmacogenes, particularly for CYP2D6, which is poorly conserved (Zhou et al., 2019; Zhou et al., 2024). Therefore, annotation and functional predictions for novel or rare variants were made with the aid of OpenCRAVAT web server (https://opencravat.org) (Pagel et al., 2020). This server includes many established variant annotation tools such as SIFT, and newer tools such as AlphaMissense (Cheng et al., 2023), which has shown superior performance for predicting impacts of variants in pharmacogenes (Zhou et al., 2024).

## 3 Results

135 individuals were successfully sequenced for *CYP2D6,* with 70 of these also sequenced for *CYP2C19.* A further 3 participants returned *CYP2C19* sequences but failed to amplify for *CYP2D6*, giving 73 *CYP2C19* genotypes in total.

In the *CYP2D6* data, *1 was the most common allele, with a frequency of 0.341, followed by *2, *4, *10 and *71 (0.233, 0.104, 0.100 and 0.100 respectively) (Table 1). This high frequency of *71 is consistent with our previously reported findings in Māori and Pacific peoples (Hitchman et al., 2022).

**TABLE 1:** *CYP2D6* Star allele frequencies and associated information.

CYP2D6 star allele	Number of haplotypes observed (n = 270)	Allele frequency: this study (n = 135)	Allele frequency: oceanian^a^ (n = 999)	Allele frequency: european^a^ (n = 65,090)	Activity score^b^	Impact
*1	92	0.341	0.617***	0.285**	1	Normal function
*2	63	0.233	0.061***	0.185**	1	Normal function
*4	28	0.104	0.018***	0.185***	0	No function
*5	4	0.015	0.035***	0.03*	0	No function
*6	1	0.004	0	0.011	0	No function
*9	2	0.007	0*	0.028***	0.5	Decreased function
*10	27	0.100	0.057***	0.016***	0.25	Decreased function
*33	1	0.004	n.d	0.01	1	Normal function
*35	6	0.022	0.004***	0.055***	1	Normal function
*41	16	0.059	0.032**	0.092**	0.5	Decreased function
*59	1	0.004	0.002	0	0.5	Decreased function
*71	27	0.100	0.043***	0***	?	Uncertain function
*108	1	0.004	n.d	0.003	?	Unknown function
*1x2	1	0.004	0.119***	0.008	2	Increased function

^a^ Allele frequencies as reported in ClinPGx (https://www.clinpgx.org/page/pgxGeneRef). “n.d.” indicates no data. Significant differences (chi-square test) between allele frequencies from this study vs. Oceanian and European are denoted thus: * <0.05; ** <0.01; *** <0.001.

^b^ Activity score and associated metaboliser phenotype as defined by Caudle et al. (2020).

For *CYP2D6*, the majority of individuals within this cohort were predicted to be normal metabolisers (54%), followed by 25% as intermediate metabolisers, however close to 20% of the cohort were unable to be assigned a metaboliser phenotype due to the uncertain function of *71 (Table 2). One individual was predicted to be an ultra-rapid metaboliser, carrying a duplication of *CYP2D6* (*1x2). In this sample, one of the *1 alleles when phased was found to carry an intronic variant (rs1208092320) previously unreported in the PharmVar *CYP2D6* database (Gaedigk et al., 2021). Four individuals carried a *CYP2D6* deletion (*5), with one predicted to be a poor metaboliser due to their genotype of *4/*5.

**TABLE 2:** Inferred metaboliser frequencies within cohort.

Inferred phenotype (from genotype)	CYP2D6		CYP2C19	
	Count (n = 135)	Frequency	Count (n = 73)	Frequency
Ultra-rapid	1	0.007	0	0
Rapid	n/a	n/a	7	0.096
Normal	73	0.541	27	0.370
Intermediate	34	0.252	33	0.452
Poor	1	0.007	6	0.082
Uncertain/unknown	26	0.193	0	0

Several alleles or suballeles were identified which were not reported in the PharmVar *CYP2D6* database (Table 3). This included two non-synonymous exonic variants, one at position 5,102 (rs377617003, R28H) and the other at position 6706 (G145C, rs1399114415). Both of these had mixed impact scores from SIFT and AlphaMissense (Table 3).

**TABLE 3:** New CYP2D6 alleles and suballeles from this study.

Variant position (*CYP2D6* reference)^a^ [GRCh38]	*SNP* rsID	Variant^b^	Number observed in cohort	Variant annotation	PharmVar name
4674 (UTR) [42131137]	n.a	T > C	1	—	*1.070
5102 (exon 1) [42130709]	rs377617003	G > A (R28H)	5	SIFT = 0.018 (deleterious) AlphaMissense = 0.122 (benign)	*4.036
5243 (intron 1) [42130568]	rs1208092320	C > T	1	Present on one copy of a duplicated *1 allele	*1.069
6706 (exon 3) [42129105]	rs1399114415	G > T (G145C)	1	SIFT = 0.001 (deleterious) AlphaMissense = 0.414 (indeterminate)	*186.001
6810 (intron 3) [42129001]	rs76326664	A > G	3	Previously identified in CYP2D6*129 and *133, identified in this sample alongside *2 core variants	*2.039
7032 (intron 4) [42128779]	rs752453767	G > A	1	—	*1.071
7265 (intron 4) [42128546]	rs936306239	C > T	2	—	*2.038
7373 (intron 4) [42128438]	rs79650744	G > A	1	—	*41.010
8428 (intron 7) [42127383]	rs28371728	T > C	2	—	*4.037
8446 (intron 7) [42127365]	rs1483955943	C > G	1	—	*4.038

^a^ Reference NG_008376.4 (LRG_303) 1 = Sequence start.

^b^ Amino acid change denoted in brackets, where applicable.

One additional intronic *CYP2D6* variant (rs76326664) has previously been described in two star alleles (*129 and *133), but the haplotype observed here also contained rs16947 (R296C) and rs1135840 (S486T) that define *2, alongside *2.001 subvariants. Therefore in this instance, rs76326664 forms a novel *2 suballele.

For *CYP2C19*, 73 of the 151 samples were successfully sequenced and assigned a star allele status. A further twelve samples returned partial sequences, but one or more amplicons covering important star defining variants failed to amplify, and thus are not included in this report. Seventy samples provided both *CYP2D6* and *CYP2C19* genotype data.

For *CYP2C19,* the most common star allele was CYP2C19*1 with a frequency of 0.610, followed by *2, *17, and *3 (with frequencies of 0.301, 0.082, 0.007, respectively) (Table 4). The majority of individuals within this cohort were predicted to be intermediate metabolisers (45%), followed by normal, rapid and poor metabolisers (37%, 10%, 8% respectively) (Table 2). There were no ultrarapid metabolisers, and no novel variants in the coding regions of *CYP2C19* were observed.

**TABLE 4:** *CYP2C19* Star allele frequencies and associated information.

CYP2C19 Star Allele	Number of haplotypes observed (n = 146)	Allele frequency: this study (n = 73)	Allele frequency: Oceanian^a^ (n = 999)	Allele frequency: European^a^ (n = 65,090)	Impact
*1	89	0.610	0.625	0.187***	Normal function
*2	44	0.301	0.147***	0.610***	No function
*3	1	0.007	0.002	0.146***	No function
*17	12	0.082	0.215***	0.057*	Increased function

^a^ Allele frequencies as reported in ClinPGx (https://www.clinpgx.org/page/pgxGeneRef). Significant differences (chi-square test) between allele frequencies from this study vs. Oceanian and European are denoted thus: * <0.05; *** <0.001.

## 4 Discussion

Following our previous work in a cohort of 202 Māori and Pacific Peoples (Hitchman et al., 2022), these additional 135 *CYP2D6* genotypes extend our knowledge of variation in this gene in Māori. We again observed a high frequency of CYP2D6*71 (rs118203758) in this population sample, highlighting its importance for further investigation to determine its impact on metabolic phenotype.

Prior studies have predicted CYP2D6*71 to be a nonfunctional allele (Tsuzuki et al., 2003; Muroi et al., 2014; Gutiérrez Rico et al., 2020), and the AlphaMissense score for this variant is 0.705, and for SIFT is 0.017, both of which imply a likely damaging effect. Furthermore, we have evidence from *in vivo* studies that this is so (manuscript in preparation). If CYP2D6*71 is confirmed to be a nonfunctional allele, then almost 41% of our study cohort would be considered intermediate metabolisers and 4% would be poor metabolisers. CYP2D6*108 (observed in one sample) is of unknown function, with the relevant variant rs202102799 (Y355C) predicted to be probably damaging by SIFT (score of 0) but indeterminant by AlphaMissense (score of 0.354). Therefore, more work will be required to understand whether this SNP has an impact on CYP2D6 function.

An early New Zealand phenotyping study with the probe drug debrisoquine identified 5% of 101 Māori participants as poor CYP2D6 metabolisers (Wanwimolruk et al., 1995). Therefore, our findings are consistent with this earlier report. We also compared the allele frequencies of our cohort with those for Oceania and European population cohorts from ClinPGx (Table 2). This showed significantly higher allele frequencies in our cohort relative to Europeans for *1, *2, and *10, and lower frequencies for *4, *5, *9, *35 and *41. The comparison with Oceania revealed extensive allele frequency differences, although Oceania is a geographic grouping of different ancestral groups with quite distinct migratory histories, so these differences are not surprising. Of the 6,954 samples in this ClinPGx grouping, only 130 are Polynesian or Māori, and all of these were reported via our prior studies (Lea et al., 2008; Larsen et al., 2015).

Our nanopore sequencing-based survey of all *CYP2C19* coding regions in this Māori cohort did not reveal any novel variants, and confirmed that CYP2C19*2 was the most prominent non-functional allele in this cohort, with a minor contribution from CYP2C19*3. The allele frequencies in our cohort are similar to those determined in two previous studies in Māori, one targeting the genotypes of *2 and *17 and the other genotyping *2 and *3 (Lea et al., 2008; Larsen et al., 2015). An early phenotyping study in Māori also identified a 7% rate of CYP2C19 poor metabolisers (Wanwimolruk et al., 1995), similar to our predicted phenotype frequency. A comparison of allele frequencies with those from ClinPGx European population cohorts showed significantly elevated frequencies in our cohort for *1 and *17, and reduced frequencies for *2 and *3. The comparison with ClinPGx Oceania is included in Table 4 for completeness, but this comparison is subject to the caveats outlined above.

Limitations of this study include that it a sample drawn largely from one iwi (tribe) in the North Island East Coast Te Tairāwhiti region (predominantly of Ngāti Porou descent), and findings may not generalise to all Māori. However, our prior work has described similar findings for *CYP2D6* in a wider Polynesian cohort that included some participants identifying as Māori (Hitchman et al., 2022). Further study in volunteers from other iwi would be useful, particularly for *CYP2C19*, to clarify whether these findings can be generalised. Second, although the nanopore sequencing methods we applied were an efficient method for charactising variation across both genes, for some of our samples we could not obtain full DNA sequence data, due to failure of one or more of the long PCR amplicons. This led to a reduced sample size for our discovery efforts. It is also possible that our nanpore sequencing method will have missed some structural variants including hybrid *CYP2D6* genes, and future research should focus on this question.

These analyses provide an effective description of variation in two of the most important pharmacogenes in a cohort of New Zealand Māori, highlighting important allele frequencies and predicting the presence of a considerable proportion of individuals with non-standard drug response phenotypes. This demonstrates the need for further studies to underpin clinical pharmacogenetic testing among New Zealand Māori.

## Data Availability

The raw sequence data is from indigenous people, and for data sovereignty reasons we cannot place this in a public repository. However, these sequence data were reviewed by the PharmVar-CYP2D6 committee, which then assigned the allele and sub-allele names that are shown in Table 3. These names are recorded in https://www.pharmvar.org/gene/CYP2D6.
