# Supplementary material for: Allelic diversity of the pharmacogenes CYP2D6 and CYP2C19 in Māori from Te Tairāwhiti, Aotearoa New Zealand
Source: Front Genet. 2026 Feb 27;17:1668409. doi: 10.3389/fgene.2026.1668409 (PMC12981722; doi:10.3389/fgene.2026.1668409)
Supplement: Supplementary file 1 [file DataSheet1.pdf]

Supplementary data:

Supplementary Table 1 Primer sequences targeting regions encompassing the *CYP2C19* exons

Coverage	Forward primer sequence (5'–3')	Reverse primer sequence (5'–3')	Approximate amplicon size (kb)
Exon 1	AGTGTTGCTGGAAACCCCAA	TATGTTGCCACCCCCATTCC	7
Exons 2 to 5	AGCAA TTGTCTGACCATTGC	TCACTGGAAGCTGCAGAACA	7
Exon 6	CATGGCATTAACTCAGCGGC	GGGAGGGAGAGAAGGGATGT	7.1
Exon 7a	CTGGTTTTGCAGTAGCCCCT	GTGTGGTGATTCCTCAGGGG	5.9
Exon 7b[1]	CTGGCCTGTGGGGAATCATT	AGGTCACCAAAGCTGATGCA	6.6
Exon 8	A TGGTCGTGCTTCAA TGTCT	GAGGGCTCTGTCCTTGCTAA	6
Exon 9	CTCCTCCCCTAAGCCCTTCT	GCAGCAAACCTACATGGCAC	3.5
Tails[2]	TTTCTGTTGGTGCTGATATTGC	ACTTGCCTGTCGCTCTATCTTC	

[1] A second pair of primers designed for exon 7, due to inconsistency of the initial primers

[2] Tailed sequences attached to the 5' end of all primer sequences, complementary to ONT barcode kit EXP-PBC096
